# Design of a Whole-Cell Biosensor Based on Bacillus subtilis Spores and the Green Fluorescent Protein To Monitor Arsenic

**DOI:** 10.1128/spectrum.00432-23

**Published:** 2023-06-07

**Authors:** Luz I. Valenzuela-García, María Teresa Alarcón-Herrera, Víctor M. Ayala-García, Marcelo Barraza-Salas, José Manuel Salas-Pacheco, Juan Francisco Díaz-Valles, Mario Pedraza-Reyes

**Affiliations:** a Department of Sustainable Engineering, Advanced Materials Research Center (CIMAV), Arroyo Seco, Durango, Mexico; b Facultad de Ciencias Químicas, Universidad Juárez del Estado de Durango, Durango, Durango, Mexico; c Instituto de Investigación Científica, Universidad Juárez del Estado de Durango, Durango, Durango, Mexico; d Department of Biology, University of Guanajuato, Guanajuato, Mexico; University of Minnesota Twin Cities

**Keywords:** green fluorescent protein, whole-cell biosensor, *Bacillus subtilis*, arsenic pollution

## Abstract

A green fluorescent protein (GFP)-based whole-cell biosensor (WCB-GFP) for monitoring arsenic (As) was developed in Bacillus subtilis. To this end, we designed a reporter gene fusion carrying the *gfpmut3a* gene under the control of the promoter/operator region of the arsenic operon (*Pars::gfpmut3a*) in the extrachromosomal plasmid pAD123. This construct was transformed into B. subtilis 168, and the resultant strain was used as a whole-cell biosensor (*Bs*WCB-GFP) for the detection of As. The *Bs*WCB-GFP was specifically activated by inorganic As(III) and As(V), but not by dimethylarsinic acid [DMA(V)], and exhibited high tolerance to the noxious effects of arsenic. Accordingly, after 12 h exposure, B. subtilis cells carrying the *Pars::gfpmut3a* fusion exhibited 50 and 90% lethal doses (LD_50_ and LD_90_) to As(III) of 0.89 mM and As 1.71 mM, respectively. Notably, dormant spores from the *Bs*WCB-GFP were able to report the presence of As(III) in a concentration range from 0.1 to 1,000 μM 4 h after the onset of germination. In summary, the specificity and high sensitivity for As, as well as its ability to proliferate under concentrations of the metal that are considered toxic in water and soil, makes the B. subtilis biosensor developed here a potentially important tool for monitoring environmental samples contaminated with this pollutant.

**IMPORTANCE** Arsenic (As) contamination of groundwater is associated with serious worldwide health risks. Detection of this pollutant at concentrations that are established as permissible for water consumption by WHO is a matter of significant interest. Here, we report the generation of a whole-cell biosensor for As detection in the Gram-positive spore former B. subtilis. This biosensor reports the presence of inorganic As, activating the expression of the green fluorescent protein (GFP) under the control of the promoter/operator of the *ars* operon. The biosensor can proliferate under concentrations of As(III) that are considered toxic in water and soil and detect this ion at concentrations as low as 0.1 μM. Of note, spores of the P*ars*-GFP biosensor exhibited the ability to detect As(III) following germination and outgrowth. Therefore, this novel tool has the potential to be directly applied to monitor As contamination in environmental samples.

## INTRODUCTION

Arsenic (As) is considered one of the most dangerous pollutants due to its high prevalence in water and soil and its ability to accumulate through the food chain (1). Pollution with this metalloid has been associated worldwide with different health risks in humans, ranging from gastrointestinal problems to severe health effects, including cancer and arsenicosis (2, 3). Contamination of groundwater with As also represents a widespread problem, as around 140 million people in 50 countries are drinkers of water containing arsenic at levels higher than 0.13 μM, which is the maximum value limit established by the WHO (4). Therefore, a larger concern lies in the fact that arsenic promotes toxicity at low concentrations and possesses organoleptic properties that are difficult to detect (5).

In nature, As is mainly found in the oxidation states of As(V) (arsenate) and As(III) (arsenite), these arsenic species form organic and inorganic compounds that include hydrides, halides, acids, sulfides, and oxides (6). In addition to its natural presence in the environment, anthropogenic activities like agriculture have increased the levels of As in soils due to the use of arsenic-based pesticides and herbicides (7). However, only a fraction of the environmental arsenic is accessible to cells, and this bioavailable fraction depends on the characteristics of the environment and the type of organism exposed (8).

The toxicity of As depends on its oxidation state; accordingly, it has been shown that pentavalent As behaving as a molecular analog of phosphate is capable of inhibiting oxidative phosphorylation and interfering with energy generation (9). On the other hand, As(III), which can enter the cell through hexose permeases or glycerol facilitators (10–13), causes toxicity after binding to the thiol groups of enzymes such as pyruvate dehydrogenase and 2-oxoglutarate dehydrogenase, affecting its function and consequently the cellular respiration process.

The oxidation state of As not only defines its toxicity but also its mobility; consistently, inorganic forms are more toxic and mobile than the organic ones, with As(III) around 10-fold more toxic to humans than As(V) and 70-fold more toxic than monomethylarsonic acid [MMA(V)] and dimethylarsinic acid [DMA(V)] (6).

The widespread arsenic pollution of water, soil, and agroalimentary crops requires constant monitoring of arsenic levels in real time. Atomic fluorescence spectrometry (AFS), atomic absorption spectroscopy (AAS), inductively coupled plasma atomic emission spectroscopy (ICP-AES), and inductively coupled plasma mass spectrometry (ICP-MS) can detect low levels of arsenic. However, these analytical techniques, which require high-cost equipment and specialized operation, are not accessible to all the world’s populations. Biosensors for monitoring As can be a reliable alternative to these sophisticated but costly approaches; biosensors are composed mainly of a module of recognition (nucleic acids, antibodies, enzymes) and a signal transducer (thermal, optical, piezoelectrical, or electrochemical) (14, 15). Most sophisticated whole-cell biosensors, which can include one or more of the cited transduction and sensing elements, have also been reported (16, 17).

Whole-cell biosensors for metals and metalloids have been developed with reporter genes encoding enzymes that lead to a visible colorimetric change, such as luciferase and β-galactosidase, or fluorescent proteins, using promoters activated by the inducing element of interest (15, 18, 19). The promoter region of the *ars* operon, which is negatively regulated by ArsR and derepressed in the presence of As, can be fused to distinct reporter genes to generate whole-cell biosensors to monitor environmental samples contaminated with this metalloid (19–21). This approach has been used in the Gram-negative bacterium Escherichia coli (15, 19–23); nevertheless, the design of biosensors employing these approaches is less described in environmental bacteria, including Bacillus subtilis. This nonpathogenic soil bacterium, classified as a generally recognized as safe (GRAS) organism (24), can be an ideal candidate for biomonitoring distinct environments contaminated with As and other pollutants. Furthermore, B. subtilis offers the advantage of generating spores which are highly resistant to a number of harsh environmental conditions, including desiccation, heat, oxidizing agents, and UV radiation (25–27).

In B. subtilis, the *ars* operon is composed of the *arsR*, open reading frame 2 (ORF2; *yqcK*), *arsB*, and *arsC* genes, which are induced by arsenate and arsenite. The *arsR* gene, as in E. coli, codes for a transcriptional regulator that represses transcription of the operon genes in the absence of arsenic (28, 29). Employing genetic and molecular tools, in this work, we describe the design of a whole-cell B. subtilis biosensor in which expression of the green fluorescent protein-encoding gene, *gfpmut3a*, is activated in response to the presence of arsenic in the environment. In samples contaminated with As, dormant spores of the B. subtilis biosensor developed here can emit a fluorescent signal ~4 h after the onset of germination. This biosensor showed specificity to monitoring As, as well as the ability to grow under concentrations of the metalloid that are considered toxic in water and soil samples.

## RESULTS

### Design and construction of an arsenic B. subtilis biosensor.

**FIG 1:**
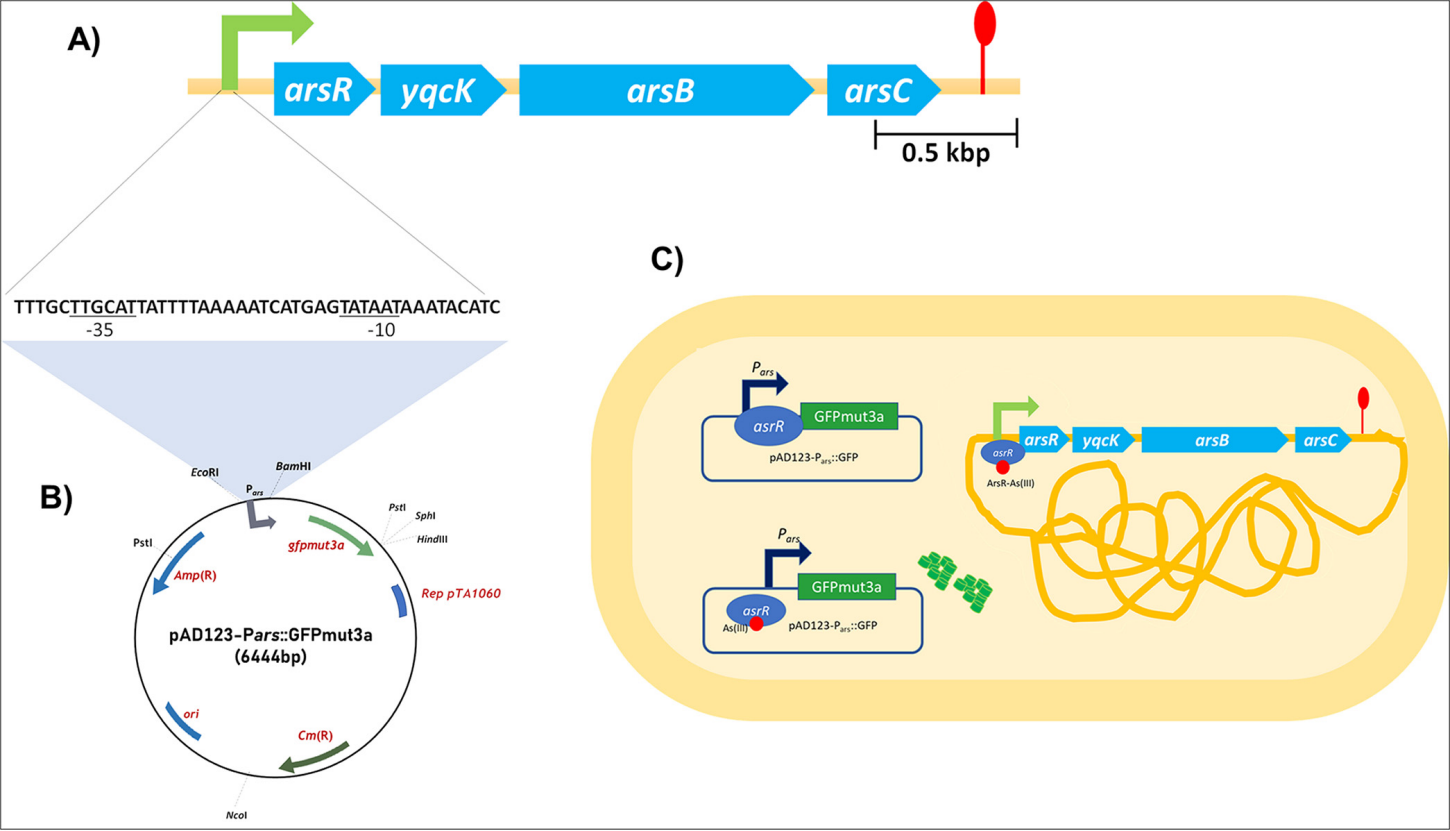
Schematic design of the whole-cell B. subtilis pAD123-*Pars::gfpmut3a* biosensor. (A) Operon *ars* in B. subtilis genome conformed by the *arsR*, *yqcK*, *arsB*, and *arsC* genes. The green arrow represents the operon promoter, highlighting the transcription binding sites, and underlining the sequences −35 and −10. The drawing is to scale. (B) A fragment of 507 bp from the −486 to +39 region of the B. subtilis *ars* operon was amplified and inserted into the vector pAD123, yielding plasmid pAD123-*Pars::gfpmut3a*. (C) Schematic representation of GFP synthesis in B. subtilis cells following derepression of plasmid pAD123-*Pars::gfpmut3a* by As(III) (red dot). A solid tangled yellow line represents the B. subtilis chromosome.

B. subtilis possesses an arsenic response operon composed of the *arsR*, *yqck*, *arsB*, and *arsC* genes (Fig. 1A) (29). The expression of this operon is under the control of the ArsR repressor, encoded by *arsR*, which is inactivated by the presence of As(III), to allow the transcription of genes that compose this operon, including *arsR* itself; *yqcK*, encoding a protein of unknown function; *arsB*, which codes for an arsenite exporter; and *arsC*, encoding an arsenate reductase (29). Taking advantage of this response to arsenic, in this work, we generated a gene construct bearing the promoter/operator region of the *arsR-yqcK-arsB-arsC* operon upstream of the *gfpmut3a* gene in the self-replicative plasmid pAD123. To this end, a 507-bp fragment, spanning from the −486 to +39 region of the *arsR* open reading frame, was amplified by PCR. As shown in Fig. 1 and Fig. S1 in the supplemental material, this fragment contains the −35 and −10 regions of the promoter as well as the operator site of the *ars* operon (29). The *ars* promoter (P*ars*) was ligated into the EcoRI and BamHI sites of the pAD123 vector, thus leaving the *gfpmut3a* gene under the control of the regulatory regions of the *ars* operon (Fig. 1B). The resulting construct was introduced by transformation into B. subtilis 168, obtaining the strain VMAG049. It was expected that As(III) in the culture medium would derepress, in the biosensor, both the *ars* operon and the *gfpmut3a* gene from the vector, thus generating fluorescent cells (Fig. 1C).

### Tolerance to As(III) of B. subtilis cells bearing a *Pars::gfpmut3a* construct.

**FIG 2:**
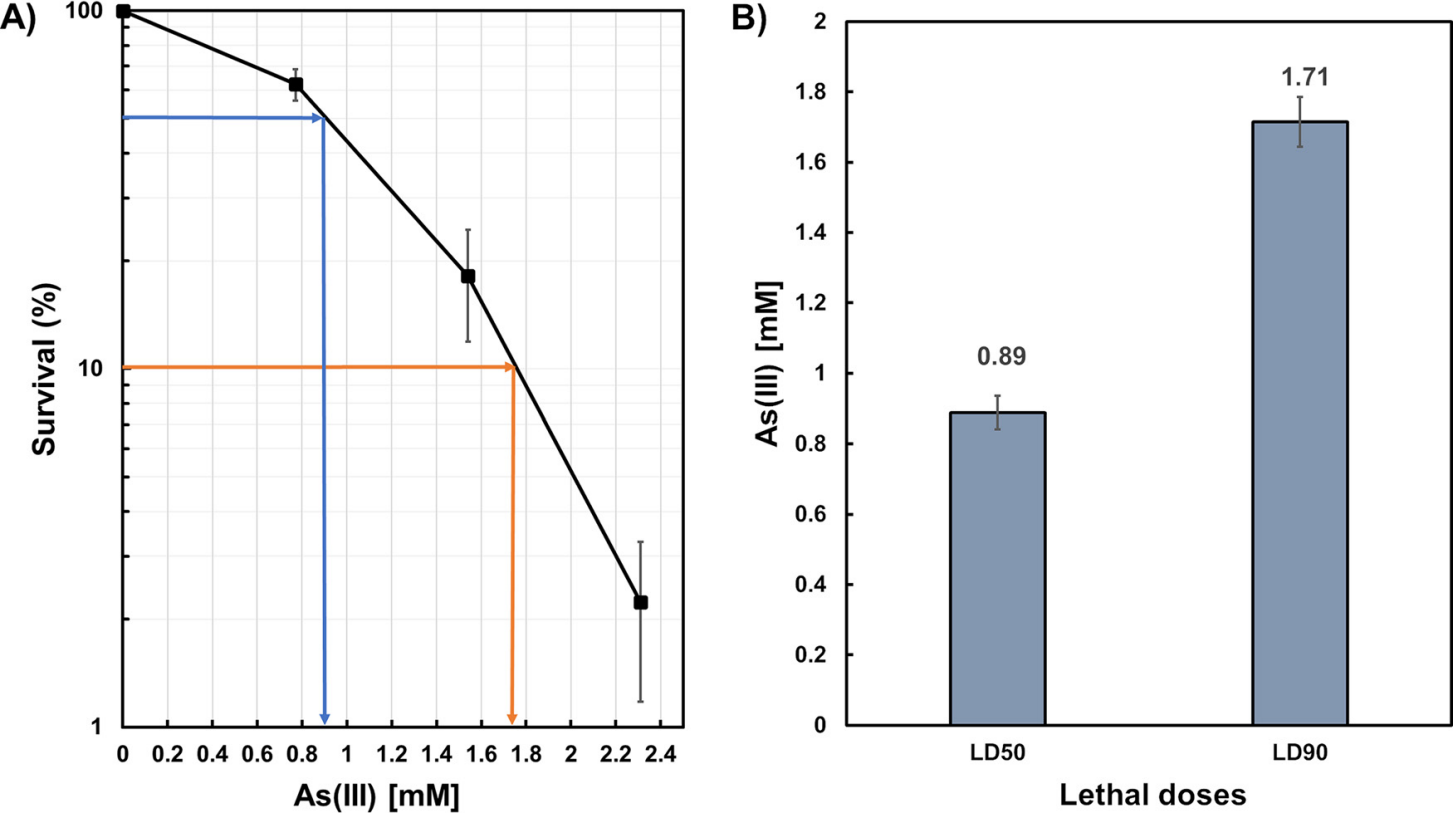
Survival of *Bs*WCB-GFP to As(III) cytotoxicity. (A) Exponentially growing cells of strain B. subtilis VMAG049 were treated with increasing doses of As(III) for 12 h, and survival was determined as described in Materials and Methods. (B) The LD_90_ and LD_50_ values were calculated from the dose-response graph in panel A. Results are expressed as the average ±SD of at least three independent experiments.

We first investigated if the growth and, consequently, the signal emitted by cells of B. subtilis carrying the *Pars::gfpmut3a* vector are influenced or not by the presence of As in the medium. To this end, the strain VMAG049, propagated to the logarithmic phase of growth, was challenged with increasing doses of As(III) for 12 h. Results revealed that cells of the arsenic biosensor were highly tolerant to the noxious effects of As(III), as they exhibited 90 and 50% lethal doses (LD_90_ and LD_50_) of 1.71 ± 0.071 mM and 0.89 ± 0.047 mM, respectively (Fig. 2A and B).

### Sensitivity to the As response of the *Bs*WCB-GFP.

**FIG 3:**
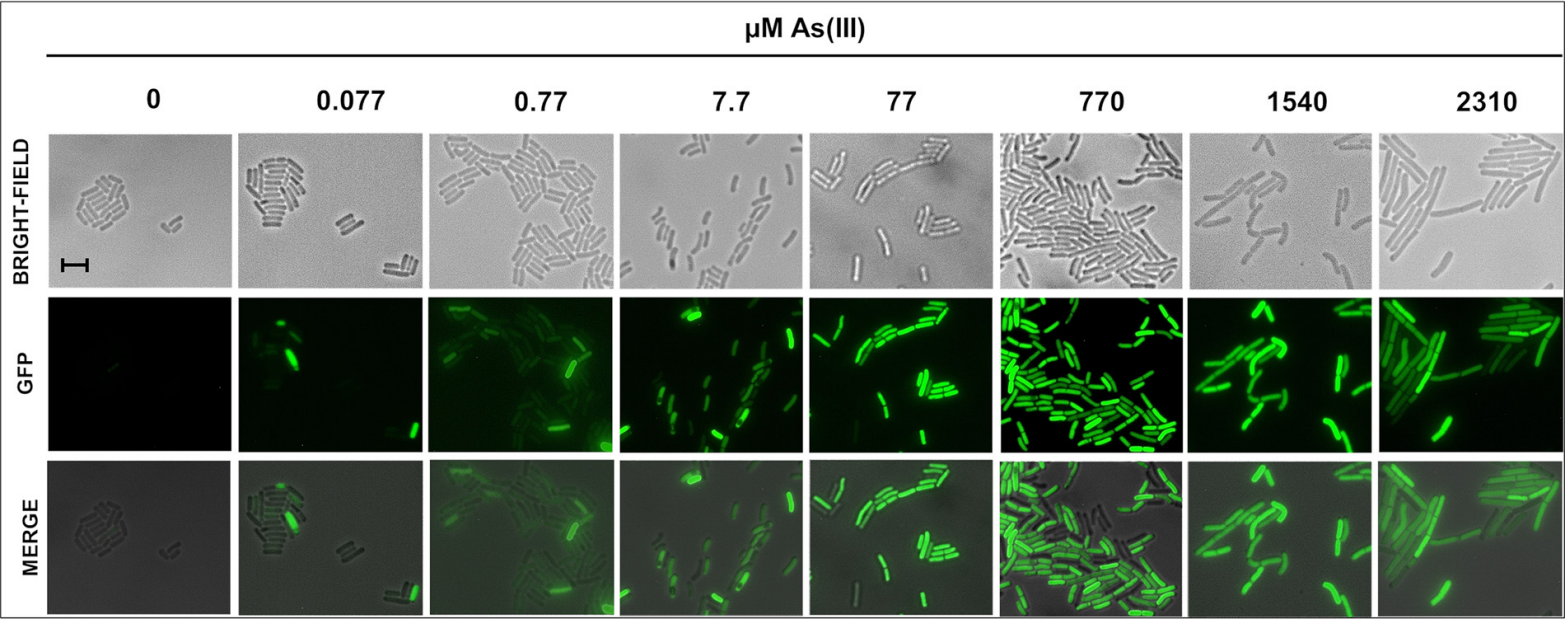
Microscopic analysis of the B. subtilis WCB-GFP strain. Cells of B. subtilis carrying the *Pars::gfpmut3a* construct collected 12 h after incubation with the As(III) concentrations indicated were observed and photographed using a fluorescence microscope at 100× magnification as described in Materials and Methods. The image shows the bright field and the fluorescence field (GFP). The overlap between the images of bright field and fluorescence is denoted as MERGE. Scale bar, 10 μm.

Employing epifluorescence microscopy, we next analyzed the range of As doses that were capable of activating the expression of the reporter fluorescent protein GFPMut3A in cells of the strain B. subtilis VMAG049. Our results revealed that *gfp* expression from the *Pars::gfpmut3a* biosensor was activated in a range of 0.077 to 2,310 μM As(III). Of note, this effect was observed even at the lower concentration evaluated, namely, 0.077 μM As, which is lower than the limit value (0.13 μM As) established by the WHO for water for human consumption (Fig. 3). Furthermore, a positive correlation among the concentration of the pollutant arsenite and the fluorescence emitted by the biosensor was observed; thus, concentrations above of 77 μM As were more efficient in inducing the expression of the GFP protein (Fig. 3).

### Selectivity of induction by arsenic ions of the *Pars::gfpmut3a* biosensor.

**FIG 4:**
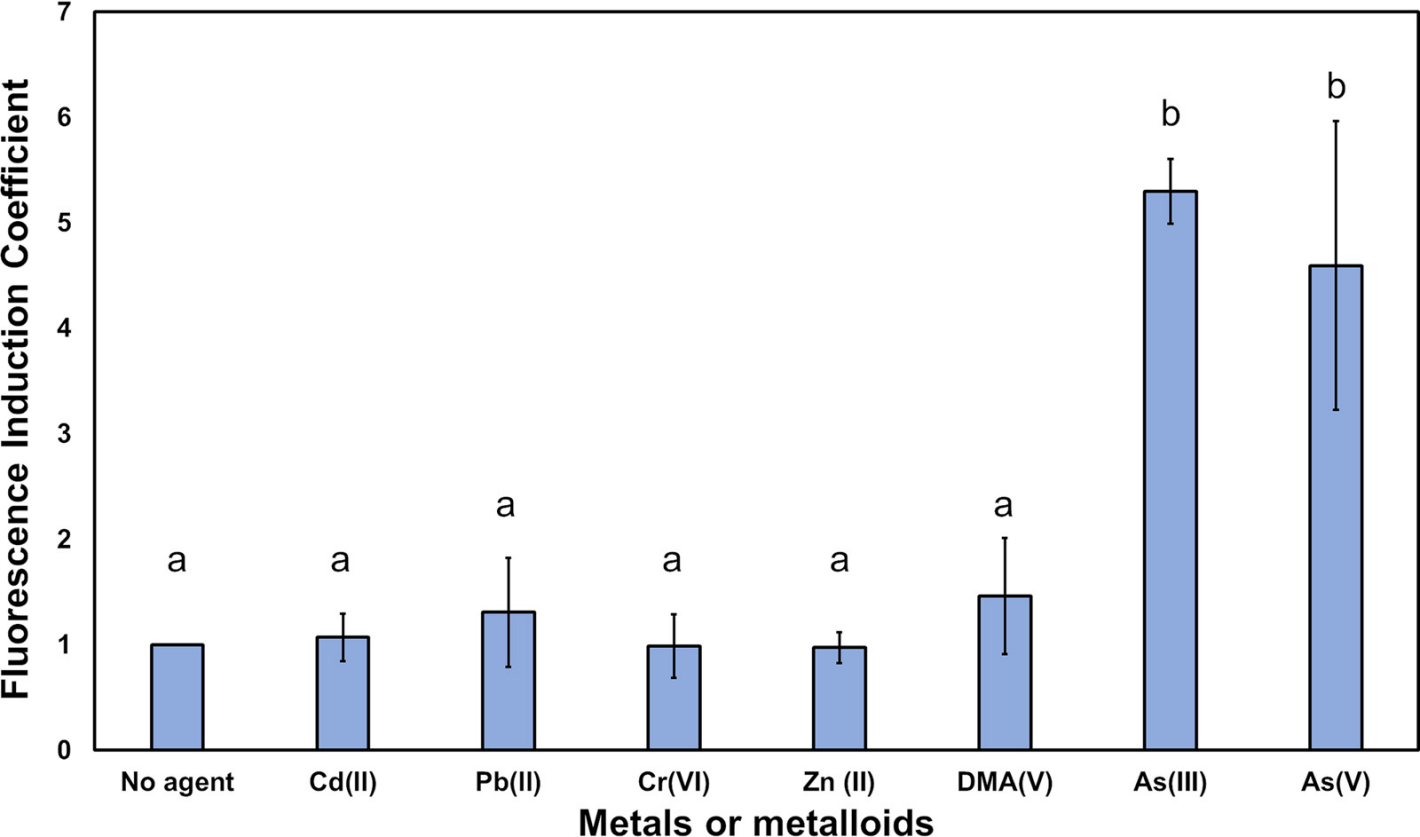
Selectivity of the arsenic B. subtilis *Pars::gfpmut3a* biosensor to distinct metal(loid). Fluorescence induction coefficient of the B. subtilis WCB pAD123-*Pars::gfpmut3a* in response to incubation for 12 h in the presence or absence of Cd(II), Pb(II), Cr(VI), Zn(II), As(III), As(V), and DMA(V). For fluorescence quantification, the Varioskan Lux plate reader was used as described in Materials and Methods. The statistical differences (a and b) between the *Bs*WCB-GFP FIC of each ion assayed were determined by ANOVA with Tukey *post hoc* test (*P* < 0.05) and are shown above each bar.

To support the application of the *Pars::gfpmut3a* biosensor to selectively monitor environmental samples contaminated with arsenic ions, B. subtilis cells bearing the *gfpmut3a* were exposed for 12 h, in addition to As(III) and As(V), the metals Pb(II), Cr(VI), Cd(II), Cu(II), and Zn(II), and the organoarsenic DMA(V) compound, all employed at a final concentration of 10 μM (see Materials and Methods). The results showed a specific response of the B. subtilis green fluorescent protein (GFP)-based whole-cell biosensor (*Bs*WCB-GFP) to both arsenite and arsenate, as the fluorescence induction coefficient values elicited by these ions were 3 to 4 times higher than those observed with other cations, whose values were in the range of autofluorescence emitted by the culture without agents (Fig. 4).

### Quantitative analysis of the fluorescence emitted by the *Pars::gfpmut3a* biosensor.

**FIG 5:**
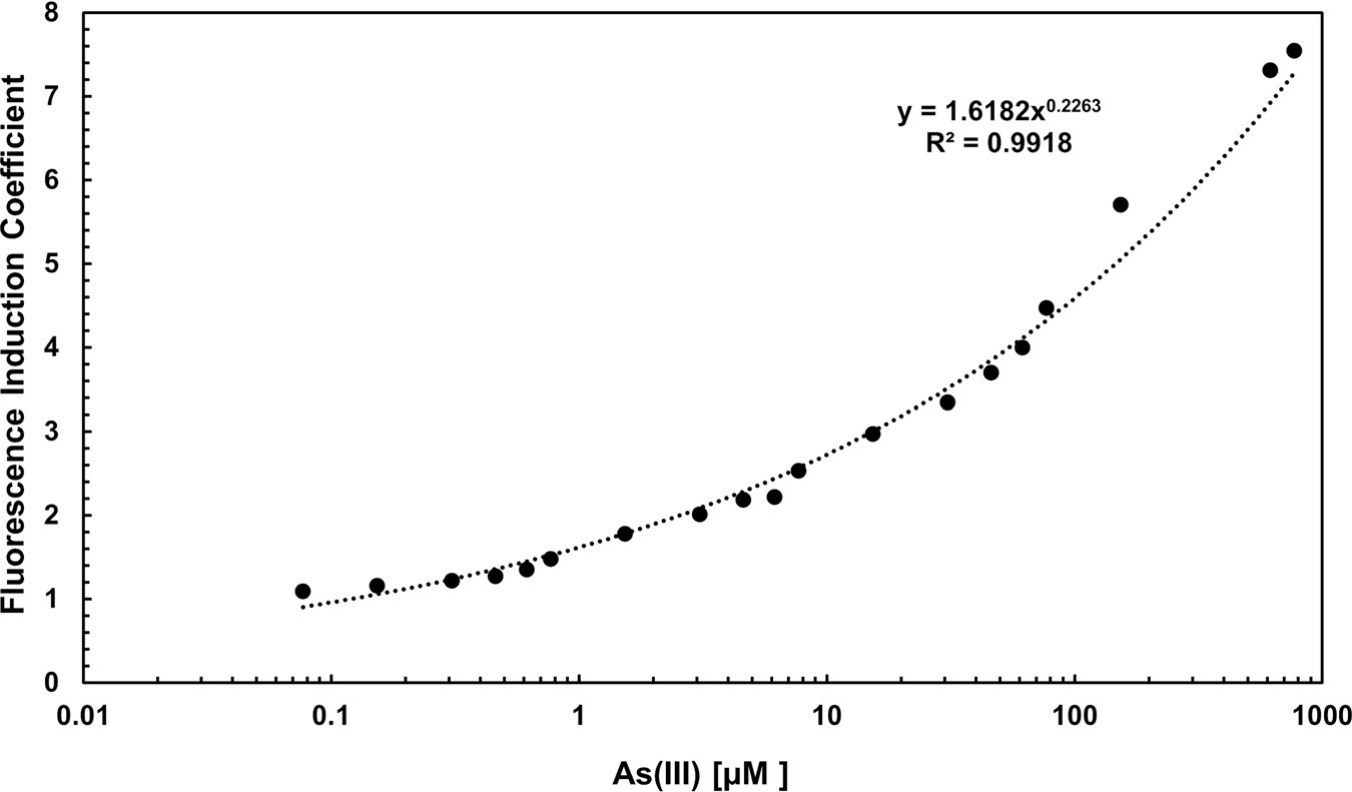
Fluorescence induction coefficient of the B. subtilis WCB pAD123-*Pars::gfpmut3a* in response to As(III). Fluorescence induction of the B. subtilis WCB-GFP was assessed by incubation for 12 h in the presence of 0 to 770 μM As(III) using a Varioskan Lux plate reader for fluorescence quantification as described in Materials and Methods.

We next proceed to determine the fluorescence levels emitted by the P*ars*-GFP biosensor as a function of the As(III) concentration. To this end, cell samples of the strain B. subtilis VMAG049 collected from the mid-logarithmic growth phase were exposed to increasing concentrations of As(III) for 12 h, and the levels of fluorescence were quantified as described in Materials and Methods. The results of these experiments, shown as fluorescence induction coefficients (FICs), revealed that the fluorescence emitted by the biosensor increased with a potential tendency from 0.077 to 770 μM As(III) (Fig. 5). Figure S2 shows the concentration in a linear scale and linear ranges of different concentration intervals; however, FIC data were best adjusted to a potential trend line represented by the equation *y* = 1.6182*x*^0.2263^ , with *R*^2^ = 0.9918 (Fig. 5). Altogether, these results indicate a quantitative response of the GFP biosensor to As(III) in a wide range of concentrations of this ion. Interestingly, similar results were obtained when these experiments were conducted with As(V) (Fig. S3).

### Determination of the fluorescence coefficient induction using spores of the *Bs*WCB-GFP.

**FIG 6:**
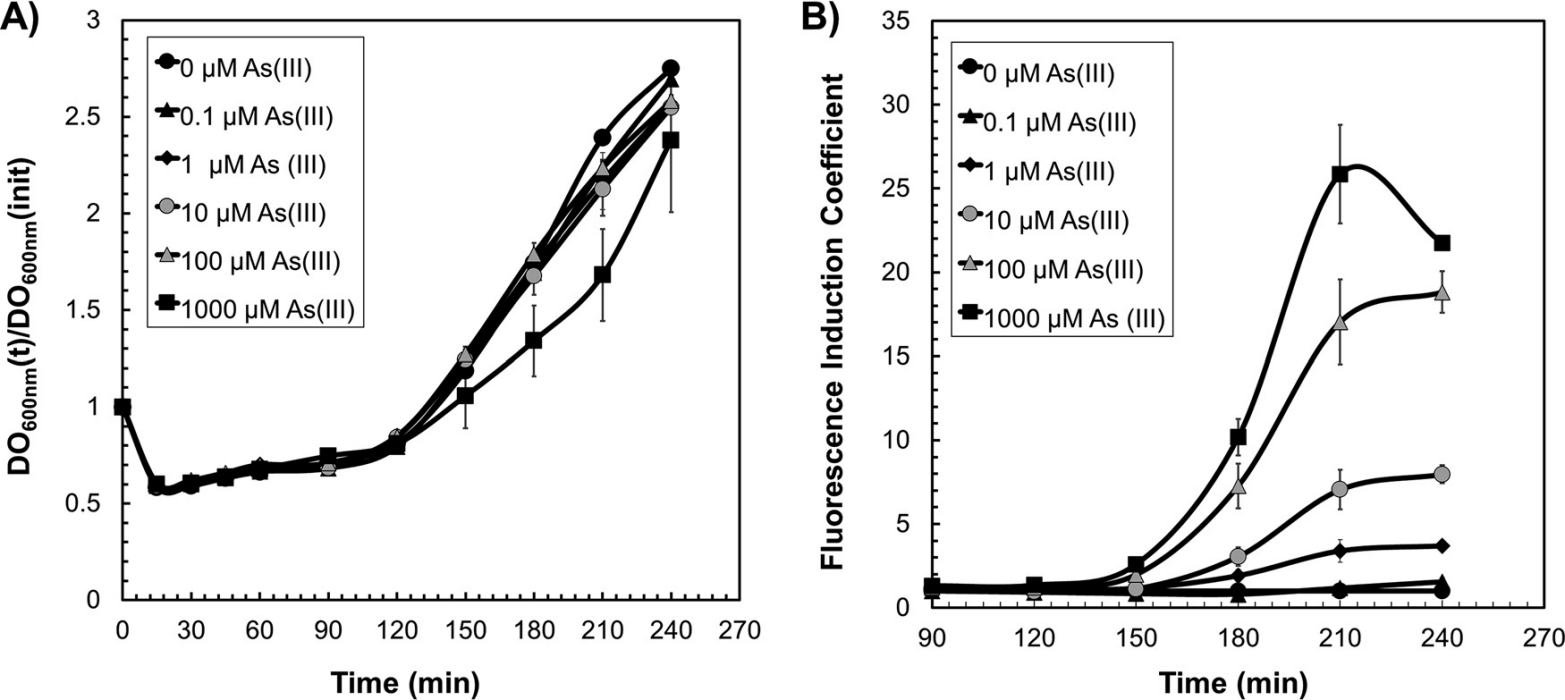
Use of spores of the *Bs*WCB-GFP for determination of FIC in response to As(III). (A) Germination-outgrowth and vegetative growth kinetics of B. subtilis spores harboring the plasmid pAD123-*Pars::gfpmut3a* in the presence of 0 to 1,000 μM As(III) added 1 h after the onset of germination. (B) Fluorescence induction coefficient of spores germinated and outgrown in the presence of different As(III) concentrations (0 to 1,000 μM). Both absorbance and fluorescence were determined using a Varioskan Lux plate reader as described in Materials and Methods. Data represent the average of three independent experiments ±SD.

Next, we investigated if the *Bs*WCB-GFP biosensor can be applied to in-field detection of samples contaminated with As. To this end, dormant spores carrying the P*ars-*GFP fusion germinated for ~1 h were incubated with increasing doses of As(III), and the fluorescence emitted by the biosensor was determined at different times. As shown in Fig. 6B, concentrations above 1 μM As(III) promoted a substantial increase in the FIC 150 to 240 min after the onset of spore germination. Furthermore, while 1 μM arsenite activated the fluorescence in the P*ars*-GFP fusion, the highest FIC value was obtained at a final concentration of 1,000 μM As(III) (Fig. 6B). Notably, only the highest concentration of As(III) (i.e., 1,000 μM) had a slightly negative effect on the germination/outgrowth kinetics of the *Bs*WCB-GFP spores (Fig. 6A). Altogether, these results demonstrated that spores harboring the plasmid pAD123*-Pars::gfpmut3a* can be used to rapidly detect, in the field, samples contaminated with As.

## DISCUSSION

The development of whole-cell biosensors is suitable for monitoring contaminants in the environment; however, this technology is not commercially available, so the development of WCB to be used in specific environments or experimental conditions is of high interest. In this work, a WCB for As detection was generated in the sporulating bacterium B. subtilis, employing the GFP-Mut3A protein as a reporter, whose induction was determined by the promoter/operator elements of the *ars* operon.

The WHO has established 10 μg/L (0.13 μM) of As(III) as the permissible limits of As in water for human consumption (2); however, this value may change among different worldwide regions (9). According to NOM 127, In Mexico, the limit for As(III) in drinkable water was recently adjusted from 0.33 to 0.13 μM (30). Regarding soil, the NOM-147 considers a limit of 22 mg/kg (22 ppm) for agricultural, residential, or commercial soil and a limit of 260 mg/kg (260 ppm) for industrial soil in Mexico (31).

The whole-cell biosensor developed here was shown to be capable of emitting a fluorescent signal in a concentration range from 0.077 to 2,310 μM (5.77 μg/L to 173.1 mg/L) of As(III); therefore, it is of potential use for the detection of arsenic contamination in water and soil. Future work will be required to establish the optimal conditions to apply this biosensor in detecting As contamination in distinct environmental samples.

Previous works have described the development of WCBs in B. subtilis for As detection, using reporter systems such as *lacZ* (32) and luciferase (33). However, the GFP-based As biosensor described in this work can directly report the fluorescence generated by the GFP-Mut3A protein, thus avoiding the use of enzymatic substrates and eliminating false positives due to cross-contamination with chemicals. Furthermore, the probability of finding fluorescent bacteria in soil and water samples for human consumption is very low; therefore, the fluorescent signal can be mostly attributed to the reported protein GFP induced by arsenic in the biosensor. It must also be pointed out that the signal emitted by GFP will not be required to induce cell lysis, thus allowing the monitoring of environmental As in real time.

The *ars* operon, which is located in the *sigK* intervening element from the genome of B. subtilis, may be a remnant ancestral element of a B. subtilis prophage (29). The transcription of this operon has been reported to be triggered by arsenate, arsenite, and antimonite (29). In parallel with these results, here, we report that the *Pars::gfpmut3a* fusion was specifically induced by arsenite and arsenate, but not by other cations like Cd(II), Pb(II), Cr(VI), and Zn(II), or by the organic form dimethyl arsenic acid [DMA(V)]. However, it remains to be investigated if other organic forms of arsenic can activate our biosensor, as phenyl arsenite [PhAs(III)] and trimethylarsine oxide (TMAO) have been shown to activate, in E. coli, the expression of the *ars* operon and an *arsR*-regulated biosensor, respectively (21). A former study revealed that a single Cys_102_-Ser change generated an ArsR protein with higher selectivity for organic forms of arsenic than for inorganic arsenic (34). These observations open the possibility in future work of widening the versatility of the arsenic biosensor described here for the detection of organic arsenic in soil samples.

A WCB for arsenic detection in B. subtilis, using *lacZ* as a reporter system, was previously described (35). In this work, the authors reported a detection limit of 7.7 μg/L (0.1 μM) for arsenite. Here, employing the GFPMut3A-based biosensor, a detection limit of 5.77 μg/L (0.077 μM) for As(III) was found. Notably, cells carrying the P*ars*-GFP biosensor that survived 12 h of incubation with 2.31 mM As(III) were able to emit fluorescence, unveiling its potential to be employed not only at low but also at high concentrations of As.

The ability to survive the noxious effects imposed by the contaminant that must be detected is a critical point to evaluate during the generation of a whole-cell biosensor. An advantage of using B. subtilis as a biosensor relies on its ability to generate spores which are highly resistant to environmental factors that are detrimental to other life forms. Therefore, employment of this bacterium will potentially allow the storage of the As biosensor for long time periods, thus reducing the risk of signal loss due to cell death (30, 32, 36). In support of these notions, it was found that after several rounds of sporulation, germination, and storage for more than 8 months, the levels of As detection in the spores with an *ars-lacZ* biosensor were not significantly affected, thus attesting to the stability of the biosensors in B. subtilis (32).

Spores can germinate and resume metabolism a short time after induction with a specific germinant (35, 37). Here, we demonstrate that germination of spores carrying the P*ars*-GFP and As(III) detection can be simultaneously measured in the same equipment (Fig. 6). These observations unveil the future development of a system that can be easily stored and transported to rapidly in-field detect and quantify As(III) in environmental samples contaminated with As(III).

Diverse WCBs for As detection with reporter genes like *lux*, *lacZ*, and *gfp* have been described in E. coli. WCB with luciferase as reporter was also generated in Staphylococcus aureus and Pseudomonas fluorescens, showing As(III) detection limits of 7.7 μg/L (0.1 μM) and 10 μg/L (0.13 μM), respectively (15), which are similar values to the detection limit value obtained in this work (5.77 μg/L = 0.077 μM). However, WCBs with better sensitivity have been reported, including those from P. fluorescens OS8 (pTPT31; *ars-lucGR*) and E. coli DH5α (*pASPW2-arsR-luxCDABE*), which exhibited detection limits of 0.77 μg/L (0.0103 μM) and 0.74 μg/L (0.0099 μM) for As, respectively (15, 38, 39). Regarding bacterial biosensors employing GFP, the E. coli pIRC140 *ars-gfp* and *ArsRCis*-*gfp* biosensors showed sensitivities of 1 μg/L (0.013 μM) and 5 μg/L (0.07 μM) for As, respectively (40, 41). Therefore, the sensitivity of our biosensor can potentially be improved, for instance, by using GFP variants with improved intensity, such as the GFPxm18 version (42). Moreover, for greater stability, reproducibility, and suitability of application in environmental conditions, it will be necessary to recombine the *Pars::gfpmut3a* biosensor into the genome of B. subtilis; this strategy also will eliminate the use of antibiotics for plasmid maintenance.

Overall, based on the advantages of sensitivity and stability under conditions of high concentrations of As, the GFP-based WCB described in this report can successfully be employed to complement traditional instrumental methods for rapid and efficient detection and quantitation of environmental samples contaminated with As.

### Conclusions.

Here, we report the generation of a GFP-based *Bs*WCB that reports the presence of As(III) in a range of concentrations that can compromise human health. The biosensor possesses a high tolerance to the harmful effects of arsenite and can be maintained active for long periods of time in the form of dormant spores. Germination, outgrowth, and As(III) detection can be simultaneously measured in a microplate reader with spores bearing the P*ars*-GFP biosensor. Therefore, the arsenic biosensor has the potential to be applied in the detection of As pollution in water and soil.

## MATERIALS AND METHODS

### Bacterial strains and plasmids, culture conditions, and reagents.

E. coli and B. subtilis strains, as well as the plasmids used and generated in this study, are described in Table 1. Lysogeny broth (LB; Lennox formulation) was used for the bacterial liquid or solid cultures. When required, chloramphenicol (Cm; 5 μg/mL), ampicillin (Amp; 100 μg/mL), and/or bacteriological agar (15 μg/mL) were added to the medium. All cultures were incubated at 37°C with agitation (250 rpm) for liquid cultures. The cell optical density (OD) of liquid cultures was monitored with the spectrophotometer Thermo Scientific Genesis set at 600 nm.

**TABLE 1:** Strains and plasmids used in this study^*a*^

Strain or plasmid	Genotype or description	Construction, source, or reference
Strains		
B. subtilis 168	*trpC2* (WT)	BGSC 1A1
E. coli DH5α	Δ(*argF-lac*)169, Δ*phoA8,* φ80d*lacZ58*(M15), *glnX44*(AS), *deoR481*, *rfbC1*, *gyrA96*(NalR), *recA1*, *endA1*, *thiE1*, *hsdR17*	Bethesda Research Laboratories
VMAG039	E. coli DH5α harboring the plasmid pJET-P*ars* (Amp^r^)	This study
VMAG045	E. coli DH5α harboring the plasmid pAD123-P*ars::gfpmut3a* (Amp^r^)	This study
VMAG049	B. subtilis harboring the plasmid pAD123 P*ars::gfpmut3a* (Cm^r^)	pVMAG045→*B.subtilis* 168; This study
Plasmids		
pAD123	Shuttle *gfpmut3a* fusion vector (Amp^r^ Cm^r^)	(44)
pVMAG049	pAD123 including the P*ars-gfpmut3a* construct (Amp^r^ Cm^r^)	This study

a X→Y, plasmid DNA “X” was used to transform strain “Y.” WT, wild type.

We formulated 10-mM stock solutions of As(V), As(III), DMA(V), and Cr(VI) formulated with Na_2_HAsO_4_·7H_2_O, NaAsO_2,_ (CH_3_)_2_AsO_2_Na·3H_2_O, and K_2_Cr_2_O_7,_ respectively. For Cd(II), Pb (II), and Zn(II), 1,000 ppm stock standard solutions of Cd(NO_3_)_2_·4H_2_O_2_, Pb(NO_3_)_2_, and Zn(NO_3_)_2_·6H_2_O from Sigma-Aldrich were used, respectively.

### Genetic and molecular biology techniques.

Genomic DNA from B. subtilis was prepared by gentle lysis with lysozyme as previously described (43). Molecular standard techniques for purification of plasmid DNA from E. coli cells, PCR, agarose gel electrophoresis, and enzymatic manipulations were accomplished according to Sambrook and Russell (34). E. coli or B. subtilis competent cells were prepared as previously described (34, 43).

### Design and generation of an arsenic biosensor construct.

To obtain a biosensor construct activated by arsenic, the plasmid pAD123 (44) was used to generate a translational fusion *Pars*::*gfpmut3a*. To this end, a region from −468 to +39 relative to the *arsR* start codon was amplified by PCR from genomic DNA of B. subtilis 168 as the template, including the −35 and −10 regions of the promoter and the operator sequence *ars* (see Fig. S1 in the supplemental material), adding restriction sites EcoRI and BamHI. The oligonucleotide primers used for the amplification of the fragment were 5′GAATTC GTG GAA ACA GTA CTT CTT 3′ (forward) and 5′ GGATCC TTC ATA TTT CCG TAG CAG 3′ (reverse) (cutting sites are indicated with underline). The PCR-amplified fragment was ligated in the pJET1.2/blunt vector and subsequently inserted into EcoRI and BamHI sites of pAD123. The generated construct (pAD123-*Pars::gfpmut3a*) was replicated in E. coli DH5α cells, and the strain was named VMAG045 (Table 1). With this plasmid, the B. subtilis 168 strain was transformed to obtain the strain used as a whole-cell biosensor, the B. subtilis strain harboring the plasmid pAD123-*Pars::gfpmut3a*, denominated as strain VMAG049 (Table 1). The correct sequence of the pAD123-*Pars::gfpmut3a* construct was corroborated by DNA sequencing with the primers 5′-GT GCCACCTGAC GTCTAAGAAA CC-3′ (forward) and 5′-CCT GTA CAT AAC CTT CGG GCA TGG-3′ (reverse) (Fig. S1). Of note, the full sequence of the plasmid pAD123 is available at https://www.lifescience-market.com/plasmid-c-94/pad123-plasmid-p-63800.html.

### Assays of sensitivity to As(III).

Sensitivities to As(III) of cells from strain B. subtilis VMAG049 (Table 1) were evaluated from dose-response curves. To this end, bacterial cultures propagated to an OD_600_ of 0.5 were split in independent aliquots (2 mL) that were amended or left untreated with As(III) to final concentrations of 0.77, 1.54, and 2.31 mM, respectively. After an incubation period of 12 h with shaking, 1 mL of each subculture was pelleted by centrifugation, washed, and resuspended in 1 mL of phosphate-buffered saline (PBS) (0.7% Na_2_HPO_4_, 0.3% KH_2_PO_4_, and 0.4% NaCl [pH 7.5]). Tenfold serial dilutions of each subculture were spread on solid LB medium, and the number of colonies that appeared after overnight incubation at 37°C were counted. The survival was calculated as the percentage of cells that survive after the exposure to As with respect to the cell survival without treatment. Experiments were repeated at least three times.

### Qualitative and quantitative fluorescence assays.

To determine the response to As, an overnight culture of B. subtilis VMAG049 was diluted 100-fold in 25 mL of fresh LB medium and propagated to an OD_600_ of 0.5. At this point, 2 mL of the culture was split into separate assay tubes and amended with increasing concentrations of As(III). After an incubation period of 12 h, 1 mL of the cultures was collected by centrifugation, and the cell pellets were resuspended in 0.2 mL of diluted 1:10 LB medium. For microscopy analyses, cell samples were immobilized in 2% agarose pads as previously described (45). Fluorescence microscopy was performed in a Leica DM2500 microscope provided with the camera Leica DFC345 FX. Images were acquired with the Leica Application Suite version 3 software. The exposure time for GFP was 0.2 s. Excitation and emission spectra were 480 and 510 nm for GFP, respectively.

To quantitate fluorescence, culture samples (1 mL) collected after 12 h exposure to increasing doses of As(III) from 0 to 770.15 μM or As(V) from 0 to 641 μM, respectively, were pelleted by centrifugation and resuspended in 1 mL of diluted (1:10) LB. Samples (0.1 mL) were transferred in triplicate to 96-well black microtiter plates. The fluorescence of the samples was determined using a plate reader, Varioskan Lux 3020-428 (Thermo Scientific), using excitation at 485 nm and emission at 510 nm filters. Growth levels in the cell samples were determined in parallel by OD_600_. These experiments were repeated at least three times per triplicate. The results were plotted as the quotient of the fluorescence over absorbance (OD_600_), and the fluorescence induction coefficient (FIC) was calculated with the following formula: FIC = [fluorescence (As)/absorbance (As)]/[fluorescence (without As)/absorbance (without As)].

### Determination of arsenic whole-cell biosensor selectivity.

To establish the selectivity of the arsenic biosensor, As(III), As(V), Cd(II), Pb(II), Zn(II), Cr(VI), and DMA(V) were added to logarithmic cultures of B. subtilis VMAG049 at a final concentration of 10 μM. After 12 h exposure at 37°C, cells were collected by centrifugation and resuspended in 1 mL of diluted (1:10) LB. The fluorescence was quantified in a plate reader as described above. At least three independent experiments in triplicate were performed.

### Detection of As with germinated spores carrying the pAD123-*Pars::gfpmut3a* plasmid.

Spores of the strain B. subtilis VMAG049 were obtained in liquid Difco sporulation medium (DSM) harvested by centrifugation, purified by washing with distilled water, and stored at 4°C until use as previously described (43). The purity of the spores was verified by phase-contrast microscopy (≥98% of spores).

The fluorescence emission of spores carrying the pAD123-*Pars::gfpmut3a* plasmid was determined as follows. Spores activated by heat shock (70°C, 30 min) and then placed in ice for 5 min were adjusted at OD_600_ of 0.5 (~75 × 10^6^ viable spores/mL) in 5 mL of GMI medium (43) supplemented with l-alanine (10 mM). Subsequently, 0.2 mL of this spore suspension was placed in 96-well flat black microtiter plates in triplicate and incubated with shaking (240 rpm) at 37°C in a Varioskan Lux 3020-428 plate reader, and the optical density of the cultures was monitored at 600 nm with readings each 0.2 s. After 1 h (the time in which the germination was completed), the plate was removed from the reader, As(III) was added at concentrations of 0, 0.1, 1, 10, 100, and 1,000 μM, and the incubation with shaking was continued in the plate reader for 3 additional hours. The absorbance (OD_600_) and fluorescence were determined in 30-min periods with measuring times of 0.2 s and were reported as described in “Qualitative and quantitative fluorescence assays.” These experiments were repeated at least three times.

### Statistical analyses.

FIC data were evaluated for normality, resulting in normal data. An analysis of variance (ANOVA) test with Tukey *post hoc* analysis was applied to calculate significant statistical differences with *P* values of ≤0.05. Statistical analyses were carried out with Minitab 18 software.

### Availability of data and materials.

All data have been shown in the paper.
