# Supplementary material for: Design of a Whole-Cell Biosensor Based on Bacillus subtilis Spores and the Green Fluorescent Protein To Monitor Arsenic
Source: Microbiol Spectr. 2023 Jun 7;11(4):e00432-23. doi: 10.1128/spectrum.00432-23 (PMC10433799; doi:10.1128/spectrum.00432-23)
Supplement: Supplemental file 1 — Fig. S1 to S3. Download spectrum.00432-23-s0001.docx, DOCX file, 0.6 MB [file spectrum.00432-23-s0001.docx]

**Supplementary material**

Design of a Whole-cell Biosensor Based on *Bacillus subtilis* Spores and the Green Fluorescent Protein to Monitoring Environmental Arsenic

Luz I. Valenzuela-García, ^a^ #María Teresa Alarcón-Herrera,^a^ Víctor M. Ayala-García,^b^ Marcelo Barraza-Salas,^b^ José Manuel Salas-Pacheco,^c^ Juan Francisco Díaz-Valles,^b^ Mario Pedraza-Reyes^d^

^a^Department of Sustainable Engineering. Advanced Materials Research Center (CIMAV) Durango, México

^b^ Facultad de Ciencias Químicas. Universidad Juárez del Estado de Durango, Mexico.

^c^ Instituto de Investigación Científica. Universidad Juárez del Estado de Durango, Mexico.

^d^ Department of Biology, University of Guanajuato, Mexico.

Running Head: *B. subtilis* spores as biosensor for arsenic detection

#Address Correspondence to Maria T. Alarcón Herrera, teresa.alarcon@cimav.edu.mx


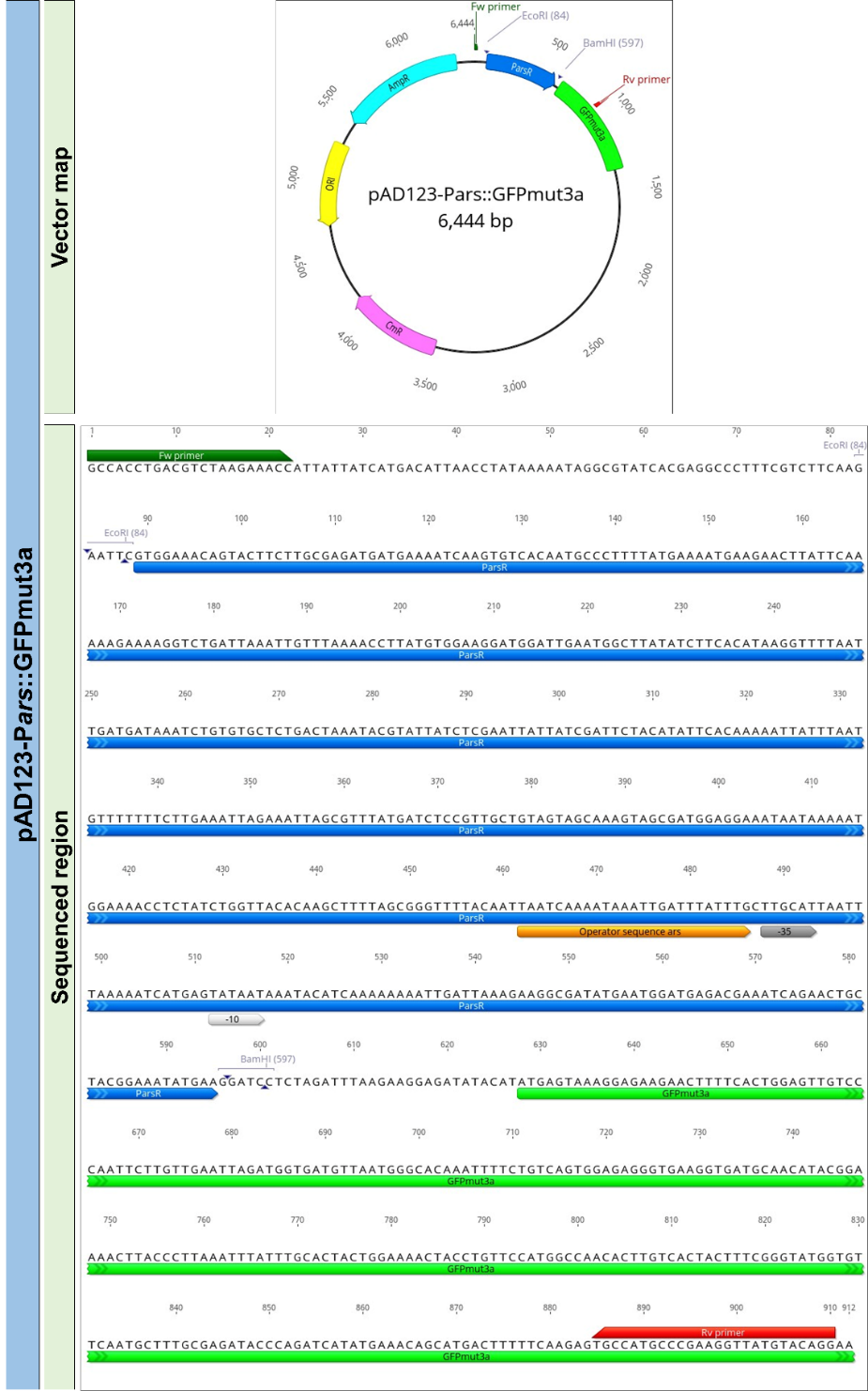


**Figure S1. Plasmid *Pars*::GFPmut3a.** The sequence of the fragment cloned into the pAD123 vector is shown in the figure. The primers used for sequencing the fragment are highlighted in green as “Fw primer” and in red as “Rv primer” (Materials and methods). In orange the operator region where the ArsR repressor binds is indicated. In dark gray and light gray, the -35 and -10 regions are indicated respectively. A fragment of the sequence of the GFPmut3a gene of the vector is indicated in green. Geneious Prime 2023.1.1 was used to generate the figure of plasmid pAD123-*Pars*::GFPmut3a and to visualize the sequenced region. (https://www.geneious.com)


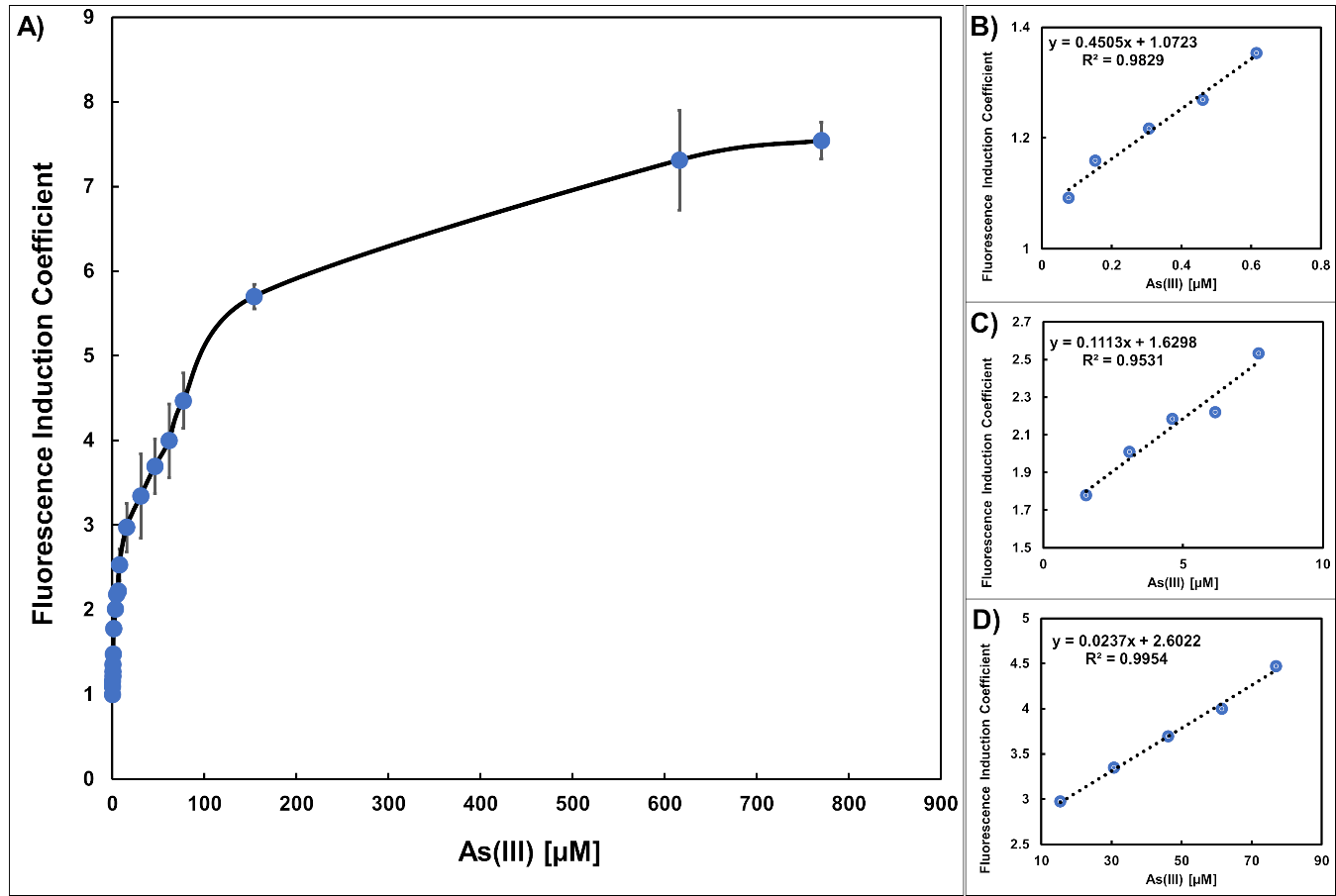


**Figure S2. Fluorescence induction coefficient of the whole-cell biosensor GFP in response to As(III) with linear scale for concentration.** Fluorescence induction of the *B. subtilis* WCB GFP was assessed by incubation for 12 hours in the presence of 0-770 μM of As(III) (A). Linear ranges are shown for 0.077-0.61 µM (B), 1.5 -7.7 µM (C), and 15-77 µM (D) As(III).


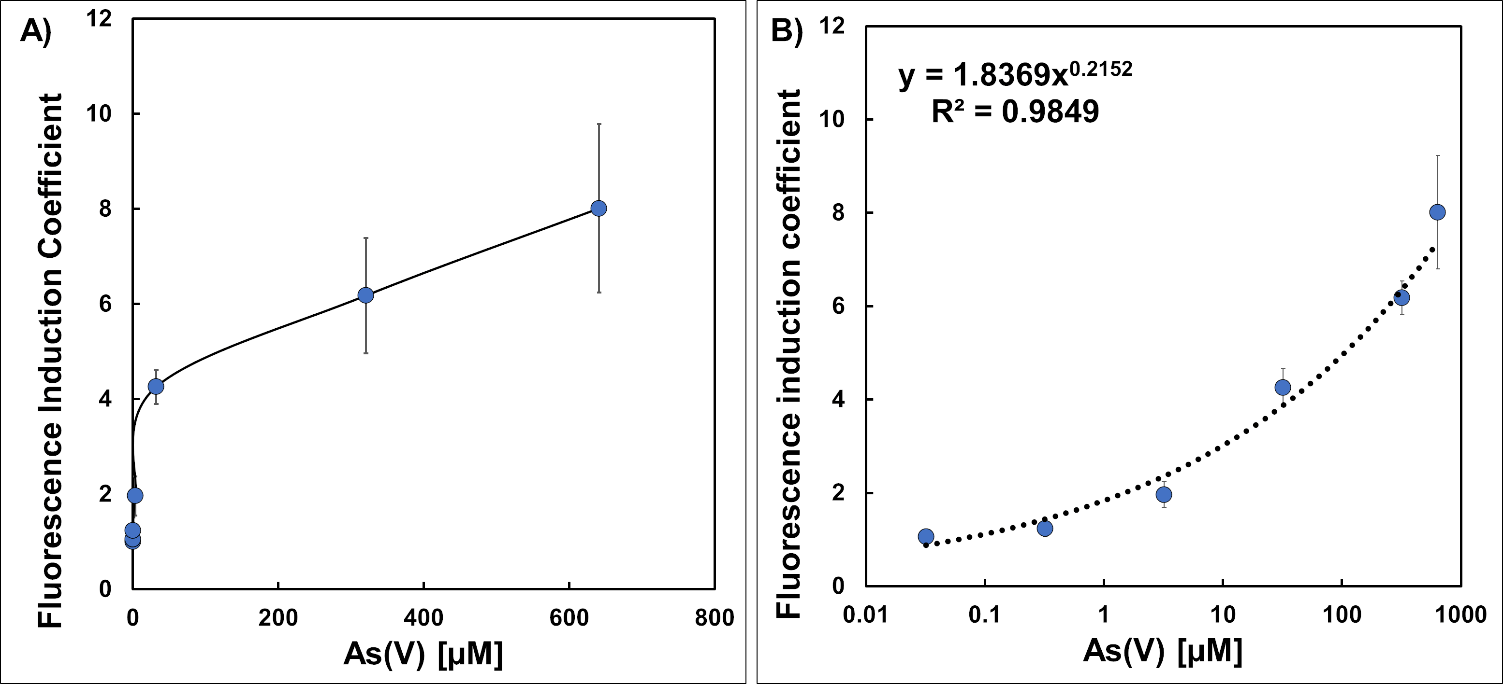


**Figure S3**. **Fluorescence induction coefficient of the whole-cell biosensor GFP in response to As(V).** Fluorescence induction of the *B. subtilis* WCB GFP was assessed by incubation for 12 hours in the presence of 0-641 μM of As(V) and graphed in linear (A) or logarithmic scale (B) for comparison.
